# Comprehensive Overview of *Candida auris*: An Emerging Multidrug-Resistant Fungal Pathogen

**DOI:** 10.4014/jmb.2404.04040

**Published:** 2024-06-15

**Authors:** Ji-Seok Kim, Hyunjin Cha, Yong-Sun Bahn

**Affiliations:** Department of Biotechnology, College of Life Science and Biotechnology, Yonsei University, Seoul 03722, Republic of Korea

**Keywords:** *Candida auris*, fungal pathogen, biology, epidemiology, virulence, drug resistance, genetic manipulation

## Abstract

The rise of *Candida auris*, a multidrug-resistant fungal pathogen, across more than 40 countries, has signaled an alarming threat to global health due to its significant resistance to existing antifungal therapies. Characterized by its rapid spread and robust drug resistance, *C. auris* presents a critical challenge in managing infections, particularly in healthcare settings. With research on its biological traits and genetic basis of virulence and resistance still in the early stages, there is a pressing need for a concerted effort to understand and counteract this pathogen. This review synthesizes current knowledge on the epidemiology, biology, genetic manipulation, pathogenicity, diagnostics, and resistance mechanisms of *C. auris*, and discusses future directions in research and therapeutic development. By exploring the complexities surrounding *C. auris*, we aim to underscore the importance of advancing research to devise effective control and treatment strategies.

## Introduction

Fungal pathogens have emerged as a significant threat to public health, affecting millions of people worldwide. Despite their critical role in ecosystems, a subset of these organisms can cause diseases in humans, ranging from superficial infections to life-threatening systemic conditions. Each year, more than 6.55 million individuals suffer from a fungal disease that poses an immediate threat to life, resulting in over 3.75 million deaths, with about 2.55 million directly caused by the fungal infection [1]. This highlights the need for comprehensive research into fungal pathogenicity, epidemiology, and the development of effective treatments.

The situation is further complicated by the emergence of novel multidrug-resistant (MDR) fungal pathogens [2]. These organisms present a significant challenge to current therapeutic strategies, as they exhibit resistance to multiple classes of antifungal drugs, often leaving clinicians with limited or no treatment options. The rise of MDR pathogens has been linked to the overuse and misuse of antifungal medications, environmental changes, and increased international travel and trade, facilitating the spread of resistant strains [3]. As such, MDR fungal infections have been recognized as a growing public health concern, necessitating urgent attention to developing new antifungal agents and diagnostic tools to effectively manage these infections.

*Candida auris* is an alarming example of an emerging MDR fungal pathogen. First reported in 2009, *C. auris* has since been isolated in over 40 countries, causing outbreaks in healthcare settings and posing a significant risk to patients with compromised immune systems [4-6]. What sets *C. auris* apart is its ability to rapidly develop resistance to all three major classes of antifungal drugs, making infections difficult to treat. Additionally, *C. auris* can survive on animate and inanimate surfaces for extended periods, leading to hospital-acquired infections and complicating infection control measures [7]. The global spread and challenging management of *C. auris* infections underscore the urgent need for coordinated international efforts to address the threat of MDR fungal pathogens.

In this review, we comprehensively summarized and discussed the clinical importance, epidemiology, pathobiological aspects, genetic manipulation methods, and diagnostic and therapeutic options in *C. auris*.

## Clinical Importance of *Candida auris*

**Fig. 1:**
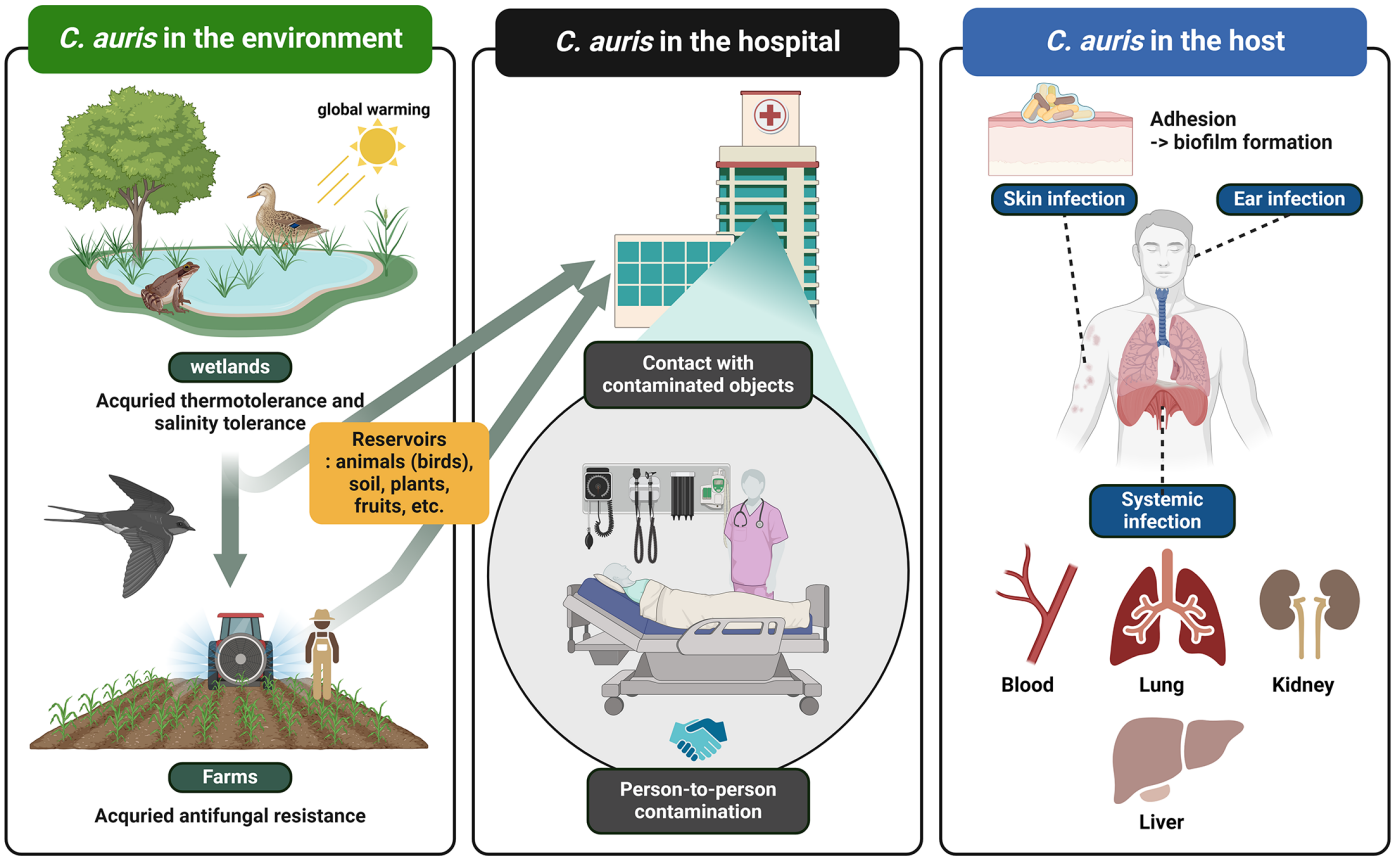
Schematic diagram of the emergence and prevalence of *C. auris*. Global warming enhances *C. auris*' thermotolerance in the environment. Exposure to various antifungal agents commonly used in agricultural practices has led to multidrug resistance in *C. auris*. The pathogen can infect both living and non-living surfaces within hospital settings. Direct contact with these surfaces facilitates rapid pathogen dissemination, subsequently resulting in illness. *C. auris* has been detected in samples from different body sites, often leading to systemic infection. Its primary mode of transmission is through nosocomial routes, particularly affecting immunocompromised patients. This figure was made using a Biorender.

The increasing prevalence of fungal pathogens poses significant challenges due to the limitations of current antifungal treatments. These limitations include severe side effects, the emergence of drug-resistant strains caused by widespread antifungal use, and a limited spectrum of activity. *Candida auris* was first discovered in 1996 by Korean researchers but was misidentified as *Candida haemulonii*. It was later revealed to be *C. auris* shortly after the Japanese group identified it as a novel fungal species in 2009 [4-6]. Initially inhabiting wetlands, *C. auris* was subsequently disseminated to rural areas via avian vectors, owing to its thermotolerance, and ultimately spread to healthcare environments in urban areas (Fig. 1). *C. auris* is commonly acquired in healthcare settings, and it has been found in various infection sites throughout the body (Fig. 1). It has been detected in urine, bile, blood, wounds, nasal passages, armpits, skin, and rectum of infected individuals [8]. In contrast to *Candida albicans*, which typically colonizes the gastrointestinal and genitourinary tracts of healthy individuals, *C. auris* is primarily known to colonize the skin [9]. However, there have been rare instances where it has been isolated from the gastrointestinal tract, oral cavity, and esophageal mucosa of infected individuals [10] (Fig. 1).

Patients with compromised immune responses, either as a result of therapeutic interventions for hematologic malignancies, bone marrow transplantation, or the use of immunosuppressive agents, exhibit a significantly increased incidence of *C. auris* infection [11]. The clinical presentation of *C. auris* infections is typically non-specific, making it challenging to distinguish from other systemic infections. In the past five years, the majority of reported cases have involved the isolation of *C. auris* from blood samples and other deep-seated infection sites [11]. Detecting *C. auris* in non-sterile body sites is crucial as its presence, even in colonized individuals, carries the potential risk of transmission, necessitating the implementation of infection control measures. Due to the fatality rate ranging from 30% to 60%, immunocompromised patients should exercise caution regarding nosocomial infection caused by *C. auris*, particularly in healthcare settings where it is associated with high mortality rates, primarily due to invasive bloodstream infection [12].

## Epidemiology of *C. auris*

**Fig. 2:**
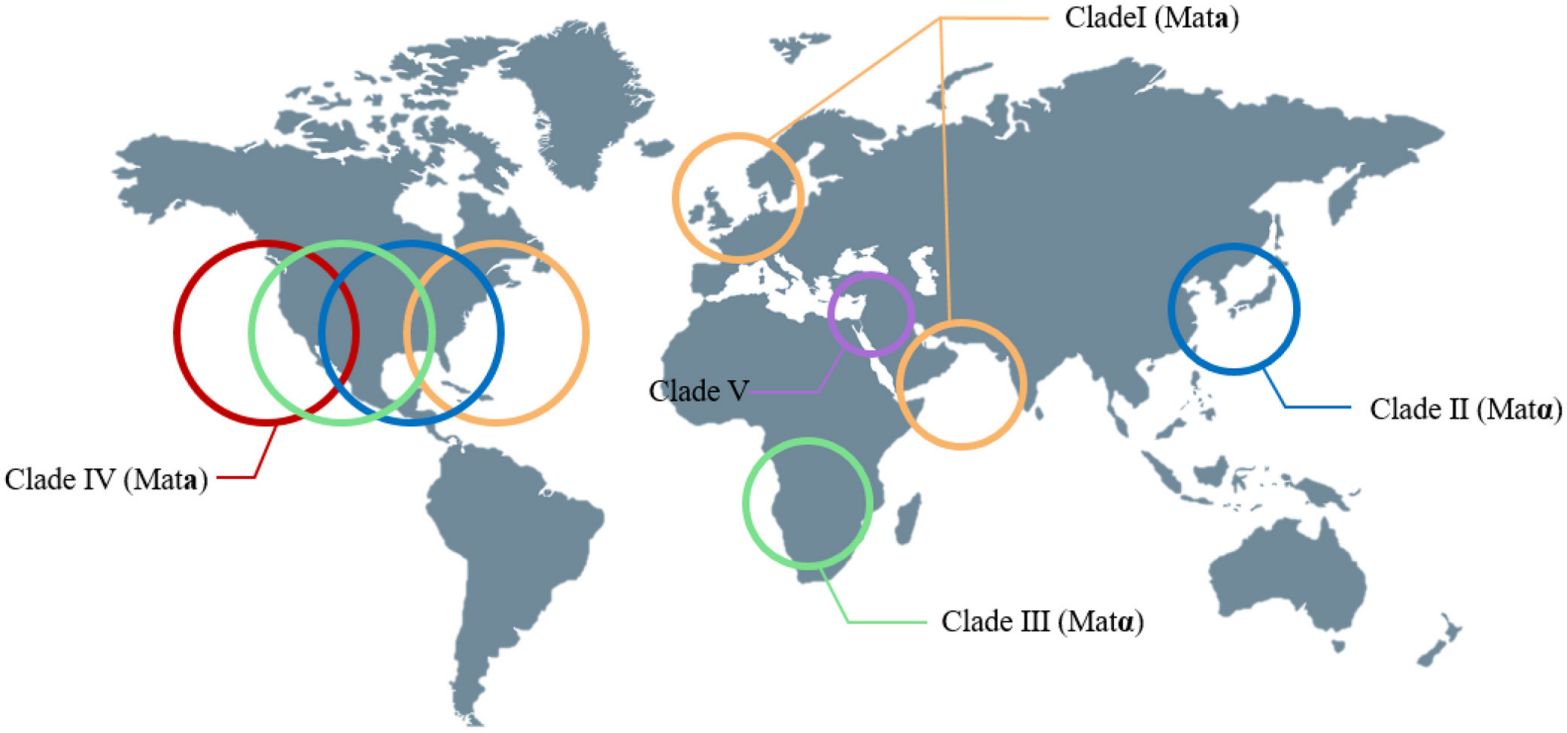
The global epidemiology of *C. auris* clades. This represents the global epidemiology of *C. auris* by clade. Each clade is marked with a circle indicating the continents where they are predominantly found, and the mating type of each clade is specified. This figure was made using a Biorender.

*Candida auris* was first documented in 2009 from the ear of a female patient in Tokyo Metropolitan Geriatric Hospital, Japan [13]. Its species name, "auris," is derived from the Latin word for "ear," reflecting its initial discovery in the ear. Based on phenotypic, chemotaxonomic, and phylogenetic analyses, *C. auris* belongs to the *Candida* genus and shares a close relationship with other uncommon species, including *Candida haemulonii* and *Candida pseudohaemulonii*. Between 2004 and 2006, fifteen *Candida* isolates were collected from ear infection patients in South Korea, initially misidentified as *C. haemulonii*. However, subsequent genomic sequencing analysis revealed that the 15 ear isolates were identified as *C. auris*, not *C. haemulonii*. Following its initial isolation, *C. auris* infections have been documented in a wide range of countries, encompassing India, Pakistan, South Korea, Malaysia, South Africa, Oman, Kenya, Kuwait, Israel, United Arab Emirates, Saudi Arabia, China, Colombia, Venezuela, the United States (US), Russia, Canada, Panama, the United Kingdom (UK), and continental Europe [14]. Genomic analysis of *C. auris* has revealed four distinct clades based on geographic origin: clade I (South Asia), clade II (East Asia), clade III (Africa), and clade IV (South America) [15] (Fig. 2). These clades exhibit genetic differences, indicating the dynamic evolution of the organism. Notably, isolates within each clade show limited genetic variation. A new potential clade V, distinct from the others and characterized by over 200,000 single-nucleotide polymorphism (SNP) differences, has recently emerged in Iran [16] (Fig. 2). It is noteworthy that clades I, III, and IV have been associated with invasive infections, nosocomial transmission, and large-scale healthcare outbreaks, while clade II has been primarily linked to ear infections.

The US reported its earliest case of *C. auris* in 2013, involving a patient who had been transferred from the United Arab Emirates. Subsequently, by February 2018, more than 250 cases of *C. auris* had been identified in regions including New Jersey, the New York metropolitan area, and Illinois [15]. In early 2019, the US experienced a total of 685 confirmed cases of multidrug-resistant *C. auris*, according to the Centers for Disease Control and Prevention (CDC). By June 30, 2019, the CDC reported 195 cases in Illinois, 126 cases in New Jersey, and 355 cases in New York, all of which were confirmed to be drug-resistant *C. auris* infections [15]. Furthermore, based on emerging data, the CDC noted a significant increase in clinical cases of *C. auris*, rising from 329 cases in 2018 to over a thousand cases in 2021 [15]. In 2021 alone, a total of 2,386 patients in the US were diagnosed with *C. auris* infection [15]. In the US, the predominant identification of *C. auris* strains was initially Clade IV. However, recent findings have revealed the presence of clades I, II, and III strains as well.

The incidence of *C. auris* infections is also increasing in Europe. The first reported case of *C. auris* infection in Europe occurred in 2009 and was linked to a patient who had traveled from India [17]. Recently, the European Centre for Disease Prevention and Control (ECDC) conducted a survey on reported cases of *C. auris* and laboratory capacity in Europe. The survey revealed that between 2013 and 2017, a total of 620 cases were reported, and four hospital outbreaks occurred in two countries [18]. A significant outbreak of *C. auris* occurred in a cardio-thoracic center in London from April 2015 to July 2016. During this period, a total of 50 cases were detected, indicating the organism's capability for rapid colonization and transmission within the healthcare environment. The outbreak was characterized by its severity and prolonged duration, highlighting the serious implications of *C. auris* within healthcare settings. The initial occurrence of invasive *C. auris* infection in mainland Europe was reported in Spain. Between April and June 2016, four patients admitted to the surgical intensive care unit of Valencia La Fe University and Polytechnic Hospital in Valencia, Spain, were diagnosed with deep-seated infections caused by this highly resistant fungal pathogen [19]. Between April 2016 and January 2017, a total of 140 patients were found to be colonized by *C. auris*, while 41 patients experienced episodes of candidemia. Among them, 5 patients developed septic metastatic complications. This outbreak is considered the largest clonal outbreak in Europe [11]. Genotype analysis confirmed that the strains involved in this outbreak are distinct from previously reported strains.

The first outbreaks of *C. auris* in South America were reported in Venezuela between March 2012 and July 2013 [11]. Initially, all isolates were identified as *C. haemulonii*, but later identified as *C. auris* through genome sequencing [11]. In Venezuela, candidemia caused by *C. auris* became the sixth most common cause of candidemia. In Colombia, sporadic cases of *C. auris* infection have been reported since 2012.

In Africa, the first cases of infection were reported in the Republic of South Africa and Kenya [11]. Between 2012 and 2013, four cases were reported in the Republic of South Africa. These strains are phylogenetically distinct from strains found in Pakistan, India, and Venezuela, but closely related to strains found in the UK.

## Biology of *C. auris*

The CTG clade encompasses pathogenic *Candida* species, including *C. albicans*, *C. tropicalis*, *C. parapsilosis*, and *C. auris*, while *C. glabrata* does not fall into this group [8]. Species within the CTG clade exhibit a unique translation of the CTG codon, resulting in serine instead of leucine [8]. Like certain *Candida* species, *C. auris* can form biofilms, undergo filamenation, and transition phenotypically between specific cell types. These characteristics are believed to contribute to its virulence, ability to tolerate antifungal treatments, and survival in various natural and host environments.

In the current ecosystem, there are approximately 1.5 to 5.1 million species of fungal organisms, with the majority of fungal species unable to survive at human physiological temperatures of 37°C and above 40°C. However, unlike most fungal species, *Candida* species can survive at these temperatures. *C. auris*, in particular, can withstand temperatures above 40°C and certain strains can even survive at temperatures exceeding 42°C [20]. Furthermore, recent studies have proposed the hypothesis that global warming may have contributed to the evolution of *C. auris* as a human pathogen. Another characteristic of *C. auris* is its high tolerance to osmotic stress compared to other pathogenic fungi, as it can survive in high salt concentrations (>10% NaCl) [20]. The ability of *C. auris* to withstand high temperatures and osmotic stress contributes to its prolonged survival on both living and non-living surfaces. This resilient fungus can persist on human skin and environmental surfaces for extended periods, with reports of survival lasting several weeks. Notably, *C. auris* exhibits a remarkable tolerance to certain commonly employed disinfectants [12].

Pathogenic *Candida* species, including *C. albicans* and *C. tropicalis*, possess the ability to undergo diverse morphological transitions [14]. These transitions can occur spontaneously or in response to environmental signals, allowing them to switch between different cell types. Notable examples in these species include the yeast-hyphal transition and the white-opaque switch. These morphological changes are pivotal for their pathogenicity and mating processes, showcasing the significance of morphological plasticity in their biology. *Candida auris*, similar to other pathogenic *Candida* species, exhibits various morphological phenotypes, although the underlying regulatory mechanisms and their functional significance remain largely unexplored. While many *C. auris* isolates predominantly exist as individual yeast cells, a subset of natural isolates can form large aggregates of pseudohyphal-like cells, wherein mother and daughter cells remain attached [21, 22]. These aggregated forms generally exhibit higher resistance to antifungal drugs compared to non-aggregating cells. However, it is noteworthy that the aggregating cells demonstrate reduced virulence in the *Galleria mellonella* infection model compared to non-aggregating cells [23]. The formation of these pseudohyphal-like aggregates in *C. auris* may be attributed to impaired cell division processes. Supporting this notion, recent research has shown that DNA damage induction and disruption of replication forks under genotoxic stress conditions promote the development of pseudohyphal-like structures in *C. auris* [24]. Additionally, it has been reported that when cultured in YPD plus 10% NaCl medium, yeast *C. auris* cells can switch to a highly elongated and pseudohyphae-like form [25]. However, factors that are known to contribute to filamentous growth in *C. albicans*, such as serum, N-acetylglucosamine (GlcNAc), and high levels of CO_2_, do not play a role in the filamentation of *C. auris*.

Biofilms are structured microbial communities that form on both abiotic and biotic surfaces, and *C. auris* exhibits a remarkable ability to form biofilms. In clinical settings, *C. auris* can form biofilms on human tissue or implanted medical devices, which are considered a major cause of nosocomial infections [8]. Moreover, the formation of biofilms by *C. auris* confers a high level of resistance to antifungal agents. Regardless of the strain or clade of *C. auris*, all possess the ability to form biofilms. During biofilm production, seven highly conserved genes (*PLB3*, *IFF4*, *PGA52*, *PGA26*, *CSA1*, *HYR3*, and *PGA7*) are upregulated [20]. Unlike *C. albicans*, *C. auris* biofilms are primarily composed of budding yeast cells. Although research on the role of *C. auris* biofilms is still limited, *C. auris* biofilms may contribute to pathogenicity in addition to drug resistance

## Genetic Manipulation of *C. auris*

**Fig. 3:**
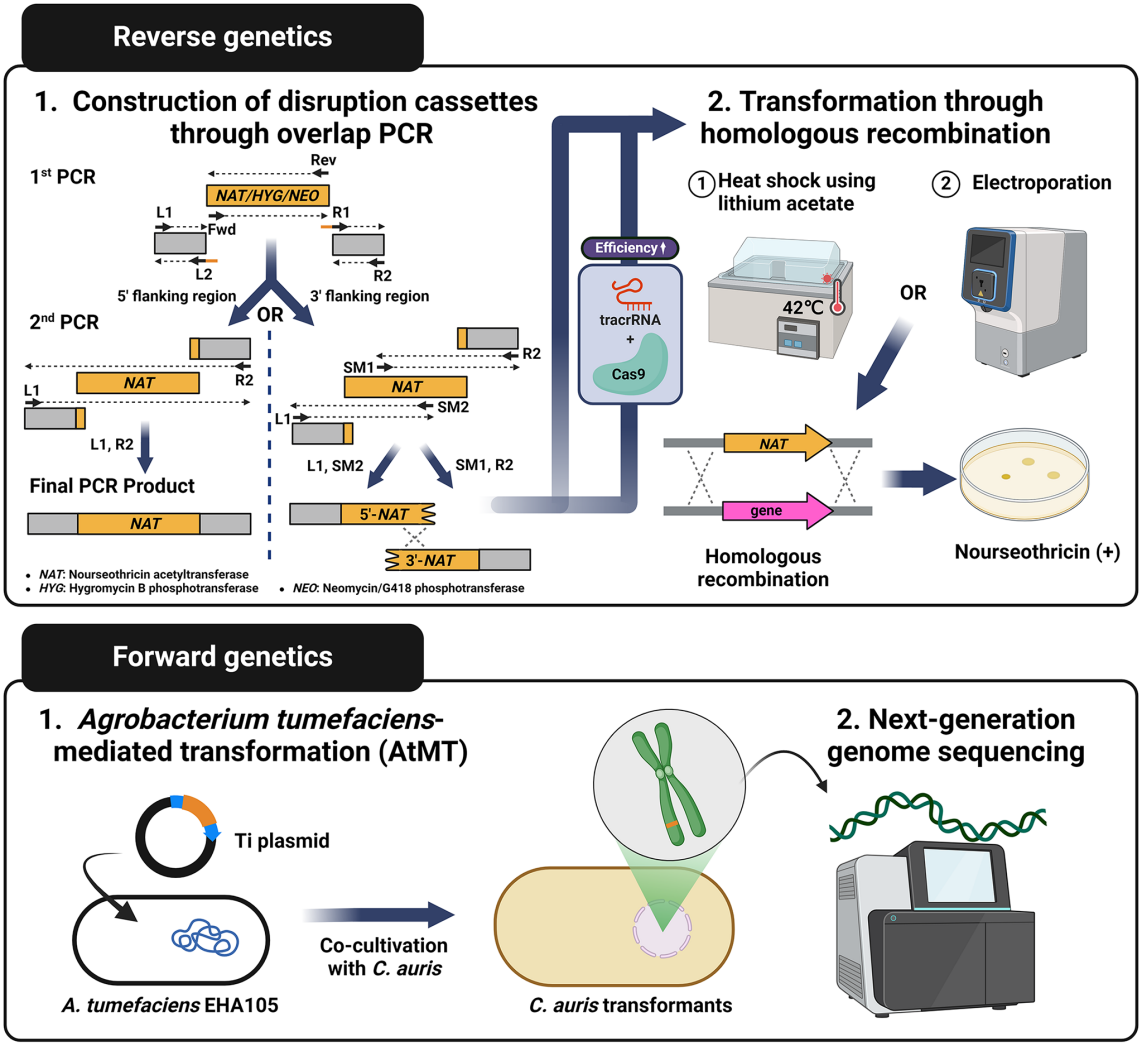
Genetic manipulation methods for *C. auris*. Both forward and reverse genetics are utilized to understand gene functions. To knock out the target gene, a disruption cassette is generated through overlap PCR. This cassette replaces the target gene with a selection marker, such as nourseothricin acetyltransferase, hygromycin B phosphotransferase, or neomycin/G418 phosphotransferase. *Agrobacterium tumefaciens*-mediated transformation (AtMT) involves incorporating a selection marker onto the Ti plasmid of *A. tumefaciens*. Co-cultivation of this genetically engineered bacteria with *C. auris* results in gene disruption by the plasmid. This figure was made using a Biorender.

Understanding the functions of genes within an organism is a fundamental aspect of molecular biology research. Both forward and reverse genetics serve as a crucial method in this endeavor. To achieve this, it is pivotal to identify and apply a suitable disruption cassette for knocking out the target gene, along with implementing efficient methods for transformation and screening. In recent studies, diverse approaches have been employed for genetic manipulation in *C. auris*. Several methodologies have been applied to genetic manipulation in *C. auris*. Due to the organism's limited recombination efficiency, previous approaches utilized disruption cassettes generated through overlap PCR, which connects approximately 1000-bp upstream and downstream flanking homology regions of the target gene to the nourseothricin-resistant selection marker (nourseothricin acetyltransferase; NAT) [26] (Fig. 3). However, following the implementation of the CRISPR-Cas9 system, the increased recombination efficiency has allowed for a reduction in the length of flanking regions to 100-200 bp [27] (Fig. 3). To introduce the disruption cassette for transformation, commonly employed methods include the heat shock method utilizing lithium acetate or the electroporation method [28, 29] (Fig. 3).

Forward genetic screens through the use of *Agrobacterium tumefaciens*-mediated transformation (AtMT) have proven successful in *C. auris* [30] (Fig. 3). In the AtMT process, a selection marker, such as NAT, is incorporated into the Ti plasmid of *A. tumefaciens*. Subsequently, this plasmid is introduced into the *A. tumefaciens* EHA105 strain, which carries the essential virulence genes for T-DNA recruitment (Fig. 3). Co-cultivation of the modified *A. tumefaciens* with *C. auris* results in gene disruption by the plasmid, conferring nourseothricin resistance. After isolating *C. auris* transformants that exhibit the desired phenotype, researchers could perform next-generation genome sequencing to identify which genes have been disrupted (Fig. 3). This methodology has been effectively employed for the identification of genes crucial to the cellular morphology of *C. auris*. Mutants leading to alterations in morphology were successfully isolated in all four clades, showing comparable rates to experiments conducted in other yeast species [31].

## Virulence and Animal Models

Research on the virulence factors associated with *C. auris* infections is still significantly lacking. Based on current understanding, morphological transition, adherence, biofilm formation, and the production of phospholipases and proteinases have been identified as virulence factors in *C. auris*.

The ability to adhere to host cells plays a critical role in microbial colonization, long-term survival, and pathogenicity. The genome of *C. auris* contains multiple orthologs of adhesins found in *C. albicans*, which are known to be involved in biofilm formation and virulence. Notably, clade II strains of *C. auris*, which are mainly associated with ear infections, exhibit notable deletions in subtelomeric regions that contain of potential adhesin genes. However, these regions remain conserved in strains belonging to clades I, III, and IV [32]. In *C. albicans*, the *ALS* adhesin gene family, including *ALS4*, is well-known for its involvement in adherence processes. Similarly, the ortholog of *ALS4* in *C. auris* shows distinct expression patterns during filamentous growth. Additionally, several other GPI-anchored cell wall genes and potential adhesins, such as *IFF4*, *CSA1*, *PGA26*, *PGA52*, and *HYR3*, were found to be upregulated during the in vitro biofilm formation of *C. auris* compared to planktonic cells [33]. Moreover, during biofilm formation, several genes, including those responsible for efflux pumps such as *MDR* and *CDR* homologs, as well as glucan-modifying enzymes crucial for the formation of the biofilm's extracellular matrix, were observed to be upregulated [33]. It was noted that inhibiting these genes resulted in increased susceptibility of biofilms to fluconazole, suggesting their significance in biofilm-mediated drug resistance mechanisms.

Lytic enzymes, including secreted aspartyl proteases (SAPs), lipases, phospholipases, and hemolysins, play a crucial role as virulence factors in fungal pathogens that infect humans [34]. These enzymes contribute to the pathogenicity of the fungi by facilitating tissue invasion, nutrient acquisition, and evasion of the host immune response [35]. *C. auris* possesses homologs of various lytic enzymes found in *C. albicans* [36]. Multiple studies have provided evidence of the lytic activity associated with *C. auris* SAPs, phospholipases, and hemolysin. These findings suggest the potential involvement of these enzymes as virulence factors in *C. auris*. According to recent research findings, the SAP activity of *C. auris* exhibits some variations depending on clades, but overall, it shows enhanced SAP activity at 37°C compared to 25°C. Moreover, *MTLa* strains secreted a higher level of SAP compared to *MTLα* strains [37].

**Fig. 4:**
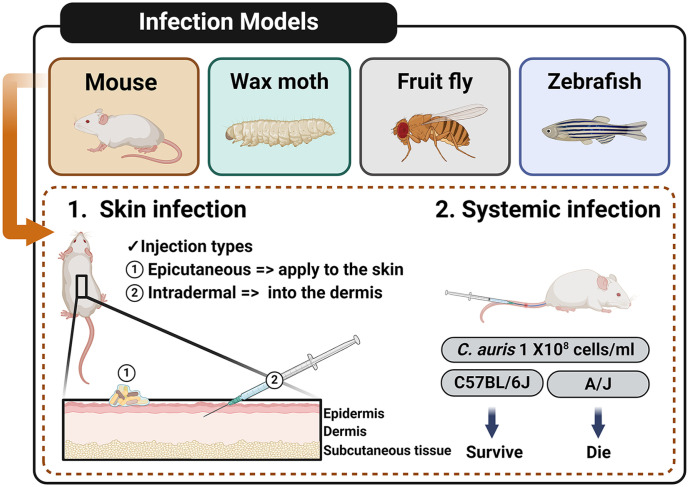
Various *C. auris* infection models. Mouse, wax moth (*Galleria mellonella*), fruit fly (*Drosophila melanogaster*), and zebrafish models are utilized for experimental assessments of *C. auris* pathogenicity. *C. auris* can induce three types of skin infections, and systemic infection outcomes vary depending on the type of mice used in the experiments. This figure was made using a Biorender.

Moreover, *C. auris* has demonstrated its pathogenicity through various infection models including mouse, wax moth, fruit fly, and zebrafish (Fig. 4). To investigate the skin colonization in the murine model, *C. auris* can be administered topically to the skin surface of the ear pinnae and shaved back, or infected into the dermis causing intradermal infection (Fig. 4). When infecting C57BL/6J female mice (8 weeks old) with a *C. auris* strain at a concentration of 10^8^ cells/ml, the mice do not succumb to the infection (Fig. 4). However, when infecting female A/J mice (8 weeks old) with the same *C. auris* strain at the same concentration, the majority of mice die within one week [38] (Fig. 4). In *G. mellonella* larvae fungal infection experiments, using an inoculum solution containing 2 × 10^7^ yeast cells of *C. auris*, *C. albicans*, *C. parapsilosis* and *C. tropicalis* isolates, *C. albicans* and *C. auris* display significantly higher virulence in terms of larval mortality kinetics and the number of larvae affected than the other *Candida* species [39]. However, *C. auris* strains exhibit reduced virulence in *G. mellonella* larvae when compared to a *C. albicans* isolate. *Drosophila melanogaster* has the potential to serve as a rapid and dependable model for studying the virulence of *C. auris* [40]. In the zebrafish model of invasive candidiasis, *C. auris* infection results in approximately 50% fewer neutrophils being recruited compared to *C. albicans* infection [41].

## Diagnosis of *C. auris*

Rapid and precise initial diagnosis is crucial in distinguishing *C. auris* infections from infections caused by other *Candida* species due to their similar clinical symptoms. In routine microbiology laboratories, the diagnosis of *C. auris* infections typically relies on culturing body fluids, blood samples, or specimens obtained from the affected sites [42]. However, accurately identifying *Candida* isolates using standard laboratory methods can be challenging. Therefore, alternative approaches such as Matrix-Assisted Laser Desorption Ionization Time of Flight (MALDI-TOF) or molecular identification through sequencing the D1-D2 region of the 28S ribosomal DNA have emerged as valuable tools [43, 44]. These methods offer enhanced accuracy in distinguishing different *Candida* species, including *C. auris*.

The Salt Sabouraud Dulcitol enrichment broth protocol is currently utilized for the isolation of *C. auris* from clinical and environmental specimens. By incorporating dulcitol as the carbon source in the broth, the growth of *Candida* species such as *C. glabrata* and *C. parapsilosis* is suppressed, except for *C. tropicalis* [45]. This protocol also employs a selective temperature of 40°C for *C. auris*. A specific *C. auris* (SCA) medium has recently been developed, which exhibits higher specificity in isolating *C. auris* by incorporating a crystal violet inhibitor into the initial medium [42]. In some clinical and healthcare laboratories, fungal cultures are screened for *C. auris* colonies using CHROMagar *Candida*. *C. auris* colonies appear as beige, white, pink, or dark purple on the agar. However, the use of CHROMagar for screening has limitations as other *Candida* species may exhibit similar morphological appearances to *C. auris* colonies. A novel chromogenic selective medium called CHROMagar *Candida* Plus has been introduced to address this issue. *C. auris* colonies on CHROMagar *Candida* Plus appear as pale cream at 35-37°C and display a distinct blue halo surrounding the colonies after 24-48 hours of aerobic incubation [42].

Conventional biochemical identification systems like VITEK 2 YST, BD Phoenix, API 20C, API ID 32C, and API 20C have restricted diagnostic capabilities, leading to frequent misidentification of *C. auris* as other closely related *Candida* species. The effectiveness of VITEK 2 in accurately identifying *C. auris* and distinguishing it from *C. duobushaemulonii* is limited [46, 47]. Initially, the reference databases of MALDI-TOF MS systems contained isolates from South Korea and Japan for *C. auris* identification. However, due to the growing number of newly discovered *C. auris* strains, regular updates of the database are essential to enhance the accuracy of *C. auris* identification. Currently, MALDI-TOF MS is widely employed as a fast diagnostic tool in clinical laboratories [43]. To ensure comprehensive identification, the reference databases must be expanded to include all phylogenetic clades of *Candida* species.

## *C. auris* Drug Resistance and Therapeutic Approach

*C. auris* is recognized as a "superbug" and poses a growing concern to human health due to its inherent resistance to one or more classes of antifungal drugs commonly used in clinical settings. The primary antifungal drug classes utilized in clinical and therapeutic practices include azoles, echinocandins, and polyenes. A comparison between the European Committee on Antimicrobial Susceptibility Testing (EUCAST) and Clinical and Laboratory Standards Institute (CLSI) methods demonstrated that *C. auris* isolates exhibit a strikingly consistent resistance to fluconazole while displaying a diverse range of minimum inhibitory concentrations (MICs) for other antifungal drug classes [48]. *C. auris* exhibits multidrug resistance to at least two antifungal classes in over 40.0% of cases, with approximately 4.0% showing resistance to all three drug classes [49]. *C. auris* has developed various molecular mechanisms of drug resistance, such as mutations in drug targets, overexpression of drug targets, and alterations in drug uptake and efflux mechanisms [50].

Azoles, the most popular class of antifungal drugs, were initially synthesized in the late 1960s. These agents exert their antifungal activity by impeding the production of ergosterol, a vital component of the fungal membrane. As a result, the growth and multiplication of the fungi are effectively inhibited [51]. The efficacy of azoles primarily relies on their ability to bind to the active site of Erg11, an enzyme involved in the ergosterol synthesis pathway. Consequently, any alterations in the active site of Erg11 due to genetic mutations can result in the emergence of drug resistance, as the binding affinity between the drug and the enzyme is affected [52]. Erg11 mutations at three specific sites (Y132F, K143R, and F126L or VF125AL) have been identified in fluconazole-resistant strains of *C. auris* belonging to distinct genetic clades [53]. These mutations are considered significant contributors to the development of resistance against fluconazole in *C. auris*. Azole resistance can also arise from the upregulation of Erg11, a phenomenon characterized by increased expression levels of the *ERG11* gene [54]. This can be attributed to either the amplification of *ERG11* itself or the upregulation of *ERG11* transcription factors, such as Upc2. Both mechanisms contribute to higher levels of Erg11, resulting in reduced susceptibility to azole antifungal agents.

Polyenes, including the well-known drug amphotericin B (AmB), are frequently employed in the treatment of *C. auris* infections. These antifungal agents exert their effects by binding to ergosterol, a vital component of the fungal membrane [55]. By forming pores in the membrane, polyenes disrupt the integrity of the fungal cell, leading to the leakage of small molecules from the cell to the external environment. This mechanism of action contributes to the antifungal activity of polyenes against *C. auris*. Changes in the sterol composition of the membrane have been identified as a mechanism of resistance. In *C. albicans*, mutations in genes such as *ERG2*, *ERG3*, *ERG5*, *ERG6*, or *ERG11* have been associated with altered sterol profiles. To investigate this phenomenon in *C. auris*, isolates from the UK displaying reduced sensitivity to AmB were examined for single nucleotide polymorphisms (SNPs) in these genes [56]. Furthermore, an increase in the expression of drug transporters has been observed following the administration of AmB. Specifically, a homolog of the *CDR6* ABC transporter showed a significant 8.7-fold upregulation in expression levels [57].

Echinocandins exert their effects by non-competitively inhibiting the activity of β(1-3) glucan synthase, a product of the *FKS1* gene [58]. This inhibition disrupts the synthesis of glucan, leading to a depletion of this essential component in the fungal cell wall. Consequently, the fungal cell becomes structurally compromised and vulnerable to osmotic stress. In *C. albicans*, resistance to echinocandins has been associated with the presence of specific mutations within two regions of the *FKS1* and *FKS2* genes [59]. These mutations have been identified as key factors contributing to echinocandin resistance. Mutations occurring at position S639 in the hot-spot 1 region of *FKS1*, specifically S639F, S639P, and S639Y, have been identified as contributing to *C. auris*' resistance to echinocandins [56]. These mutations reduce the enzymés sensitivity to the drug, and among them, S639P in *FKS1* is the most prevalent mutation associated with echinocandin resistance. *FKS2*, present as a single copy in the genome of *C. auris*, does not exhibit any mutations linked to echinocandin resistance.

**Fig. 5:**
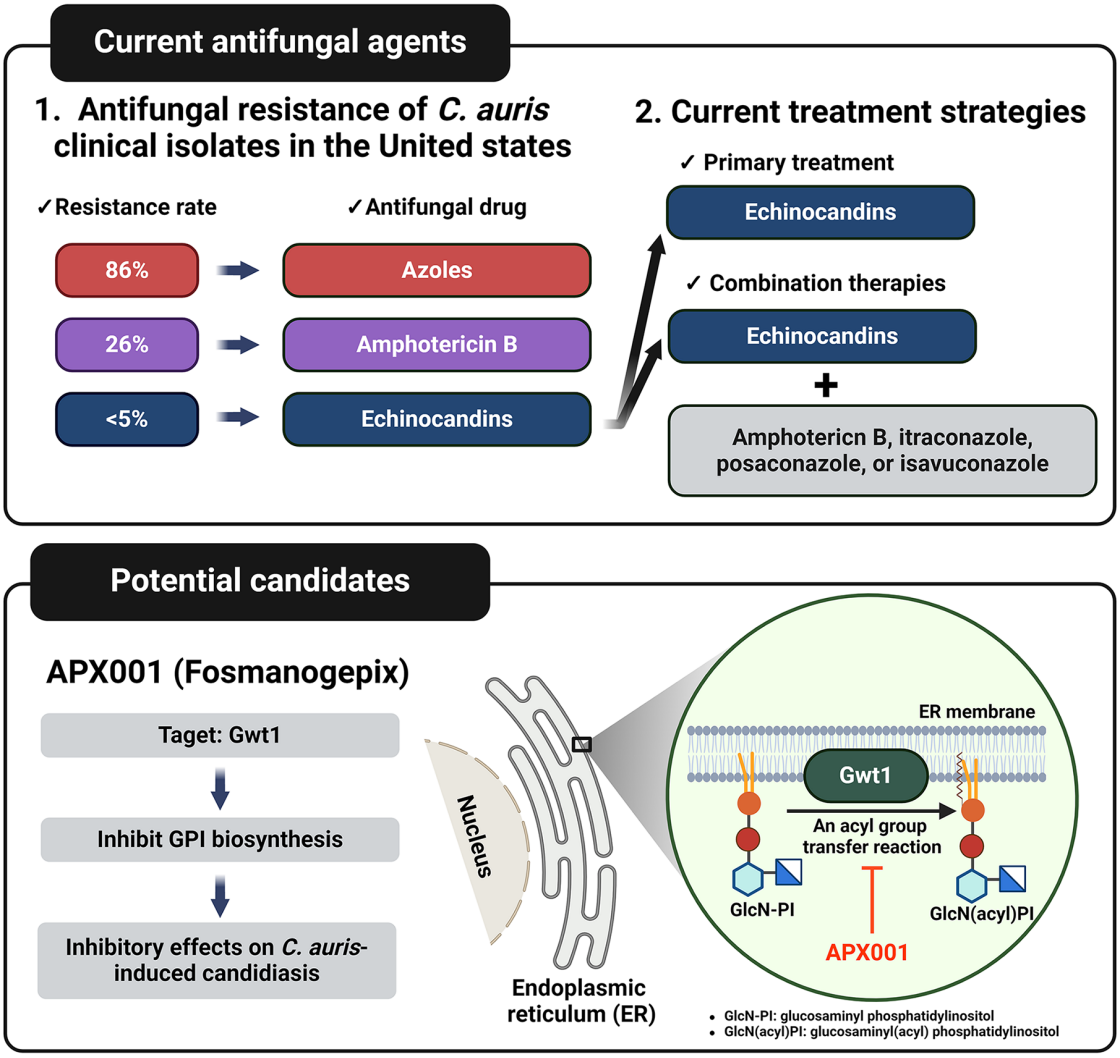
Antifungal resistance of *C. auris* and candidiasis Treatment. 86% of *C. auris* isolates show resistance to azoles, while 26% are resistant to amphotericin B. Due to low levels of echinocandins resistance, initial treatment typically involves echinocandins use. Combining echinocandins with other antifungal agents like amphotericin B, itraconazole, posaconazole, or isavuconazole has been suggested. APX0001, targeting Gwt1 to inhibit GPI biosynthesis, shows promise as a novel antifungal medication against candidiasis. This figure was made using a Biorender.

The CDC’s Antimicrobial Resistance Laboratory (Ab Lab) Network tested the resistance rate of 1294 *C. auris* isolates in 2020. The resistance to azoles was seen in 86% of isolates, and resistance to AmB was found in 26% of isolates. In contrast, the resistance to echinocandins is below 5% [60] (Fig. 5). As resistance to azoles and AmB continues to increase, echinocandins are now being widely used as the primary treatment for *C. auris* infections. However, the rise in *C. auris* strains carrying *FKS1* mutations, which confer resistance to echinocandins, has led to the recommendation of combination therapies involving AmB, itraconazole, posaconazole, or isavuconazole [61]. Nevertheless, the emergence of approximately 4% of *C. auris* strains that exhibit resistance to all currently approved antifungal drugs highlights the urgent need for the development of novel antifungal agents. Among the potential candidates, APX001 (Fosmanogepix) shows promise as a broad-spectrum antifungal agent that targets Gwt1, a protein involved in the glycosylphosphatidylinositol (GPI) biosynthesis pathway (Fig. 5). Numerous studies have demonstrated the inhibitory effects of APX001 on *C. auris*-induced candidiasis [62].

## Future Perspective

Due to its status as an emerging pathogenic fungus, *C. auris* remains an underexplored area compared to other pathogenic fungi. Extensive research has been conducted on *C. albicans*, a representative pathogenic fungus, covering approximately 30% of its 6,354 genes, totaling 1,855 genes. In contrast, research on *C. auris* is limited, with fewer than 100 genes studied among its 5,327 genes. Hence, it is crucial to explore the pathobiological functions of genes composing the genome of *C. auris*.

Acknowledged as a "superbug," *C. auris* raises growing concerns for human health due to its intrinsic resistance to one or more classes of antifungal drugs commonly utilized in clinical settings. Over the past several years, both forward and reverse genetic analyses, accompanied by in vivo virulence assays using systemic and skin infection models in animals, have identified several potential drug targets for treating *C. auris* infections and related diseases, as summarized in Table 1. Among these targets, some are evolutionarily distinct from their human counterparts or are unique to *C. auris* or fungi and absent in humans. These evolutionarily divergent or *C. auris*-specific targets warrant further investigation for the development of novel antifungal drugs.

Studies focusing on central fungal pathobiological signaling pathways, such as the cAMP-dependent protein kinase A (PKA) pathway, calmodulin/calcineurin pathway, target of rapamycin (TOR) pathway, Hog1 mitogen-activated protein kinase (MAPK) pathway, unfolded protein response (UPR) pathway, and Rim101/PacC pathway, are imperative. Research endeavors should concentrate on elucidating the correlation between these signaling pathways and the phenomena of drug resistance and pathogenicity in *C. auris*.

Recent comprehensive research on the cAMP/PKA signaling pathway in *C. auris* has uncovered a remarkable deviation from traditional models of fungal pathogenicity, where instead of inactivation, hyperactivation of this pathway leads to a significant decrease in virulence in a systemic infection model [63, 64]. This distinctive characteristic of *C. auris*, diverging from other pathogenic fungi, suggests that hyperactivation impedes proper glycogen accumulation, compromising yeast cell survival within the host and consequently reducing pathogenicity. The findings indicate that the cAMP/PKA signaling pathway contributes to *C. auris*'s virulence in a manner distinct from that observed in other pathogenic fungi, highlighting the urgent need for further investigation into alternative signaling networks and their impact on the pathobiological functions of *C. auris*.

## Figures and Tables

**Table 1:** List of genes associated with the pathogenicity of *C. auris*.

Gene name	Description	Infection method	Virulence	Reference
*HOG1*	MAP kinase activity	Systemic infection	Strongly attenuated	[65]
*PMR1*	Involved in cell wall mannosylation	Systemic infection	Weakly attenuated	[66]
*VAN1*	Involved in cell wall mannosylation	Systemic infection	Weakly attenuated	[66]
*DINOR*	Modulating genome integrity, cell filamentation	Systemic infection	Strongly attenuated	[28]
*BCY1*	Protein kinase A regulatory subunit	Systemic infection	Weakly attenuated	[64]
*ELM1*	Involved in the regulation of cell morphology	Systemic infection	Strongly attenuated	[30]
*PDE2*	Involved negative regulation of cAMP-mediated RAS signaling	Systemic infection	Moderately attenuated	[63]
*SAPA3*	Primary aspartic-type endopeptidase	Systemic infection	Moderately attenuated	[67]
*SCF1*	Adhesin specifically required for adhesion in *C. auris*	Systemic and skin infection	Strongly attenuated	[68]
